# The Cancer Tracking System (CATSystem): Study protocol of a randomized control trial to evaluate a systems level intervention for cervical cancer screening, treatment, referral and follow up in Kenya

**DOI:** 10.1371/journal.pone.0318941

**Published:** 2025-02-18

**Authors:** May Maloba, Sarah Finocchario-Kessler, Catherine Wexler, Vincent Staggs, Nicodemus Maosa, Shadrack Babu, Kathy Goggin, David Hutton, Gregory Ganda, Hilary Mabeya, Elise Robertson, Natabhona Mabachi

**Affiliations:** 1 Global Health Innovations, Nairobi, Kenya; 2 Department of Family Medicine and Community Health, The University of Kansas Medical Center, Kansas City, Kansas, United States of America; 3 International Drug Development Institute, Raleigh, North Carolina, United States of America; 4 Department of Psychology, San Diego State University, San Diego, California, United States of America; 5 School of Public Health, The University of Michigan, Ann Arbor, Michigan, United States of America; 6 Ministry of Health – Kisumu, Kisumu, Kenya; 7 Gynocare Womens and Fistula Hospital, Eldoret, Kenya; 8 The DartNet Institute, Aurora, Colorado, United States of America; PLOS: Public Library of Science, UNITED KINGDOM OF GREAT BRITAIN AND NORTHERN IRELAND

## Abstract

**Background:**

Cervical cancer (CC) is preventable, yet remains a significant public health threat, particularly in Sub-Saharan Africa. Despite considerable awareness, screening rates for CC in Kenya are low and loss to follow-up following treatment for premalignant cervical lesions remains high. This study investigates the efficacy of the Cancer Tracking System (CATSystem), a web-based intervention, to improve CC screening and treatment retention.

**Methods:**

A matched, cluster randomized controlled trial will be conducted in Kenyan government hospitals (n = 10) with five intervention and five standard-of-care (SOC) sites. The primary outcome is the proportion of women with a positive screen who receive appropriate treatment (onsite or referral). Secondary outcomes include CC screening uptake among all women and timeliness of treatment initiation. We will utilize mixed methods to assess intervention feasibility, acceptability, and cost-effectiveness.

**Discussion:**

The CATSystem has the potential to improve CC care in Kenya by leveraging existing technology to address known barriers in the screening and treatment cascade. This study will provide valuable evidence for potential scale-up of the intervention.

## Introduction

With early screening and treatment of pre-cancerous lesions, cervical cancer (CC) is preventable; yet, it is still the fourth most diagnosed cancer, globally [1]. CC disproportionately affects women in sub-Saharan Africa as access to healthcare and screening programs are still limited. In 2022, an estimated 660,000 new cases were diagnosed and 350,000 deaths occurred worldwide [1]. Over a third of these deaths occurred in Sub-Saharan Africa even though only 14% of the world’s female population lives in the region [2]. In Kenya, CC accounts for 80% of female reproductive-tract cancers [3]. Around 5,250 new cases of CC are diagnosed annually, and 3,286 women die of CC every year [4]. Women living with HIV are particularly vulnerable to CC as HIV infection is associated with increased risk of human papillomavirus (HPV) infection [5,6] and greater incidence and progression of cervical intraepithelial neoplasia (CIN) [5,7]. To achieve population level health gains, the WHO recommends countries reach 70% coverage with screening and treatment.

Although awareness of CC screening amongst Kenyan women is high, uptake of screening and treatment remains low; only 16% of women 18–69 years of age have received effective CC screening [8–12]. Key challenges to screening include lack of trained personnel, limited access to screening services outside of HIV care, as well as complex and often inadequate referral systems. While Kenya advocates for a “screen and treat” protocol, only 22%–39% of women with precancerous lesions receive and adhere to timely treatment [13]. The main modality for immediate treatment (lesion elimination) is cryotherapy techniques, which mid-level providers can be trained to perform [9]. Key challenges to implementing an effective screen and treat protocol are inadequate staffing, a limited number of trained personnel and/or insufficient training, inadequate screening spaces, and supply challenges [9] Cases with more advanced cervical lesions face additional barriers to timely treatment. These cases are referred to provincial or referral-level hospitals for loop electrosurgical excision procedure (LEEP). Treatments for invasive CC (chemotherapy, radiation, radical hysterectomy) are referred to the highest tier referral hospitals – Moi Teaching and Referral Hospital (MTRH) in Eldoret (western Kenya) and Kenyatta National Hospital (KNH) located in the capital Nairobi.

While a majority (75%) of women with invasive CC present with advanced stage disease, only 13.9% of these women start treatment with curative intent, with even fewer completing a curative treatment regimen with over 40% lost to follow-up [14]. Barriers that make it difficult for patients to navigate and access care include inefficient and complex referral networks, lack of decentralized treatment facilities causing a high burden in travel costs and time, high cost of treatment, and limited coverage by the National Health Insurance Fund (NHIF). Web-based tracking systems and eHealth interventions can help overcome many system-level challenges to increase health access in low-resource settings [15] and improve clinical outcomes, patient follow-up and adherence, and health communication [16]. Necessary eHealth infrastructure is widely available in Kenya (nearly 100% access to commercial wireless signal and 90% internet penetration) [17,18] and can be leveraged to address many of the key barriers [16,19–21].

Our intervention, the Cancer Tracking System (CATSystem), harnesses the availability of wireless technology in Kenya to improve the provision of guideline adherent cervical cancer prevention and treatment Algorithm-driven alerts aim to increase coordination between key providers across departments (Maternal and Child Health/Family Planning (MCH/FP), Comprehensive Care Center (CCC), and laboratory) by serving as a single, linked medical record for a patient’s CC care. It can extend the reach of expert providers into remote areas. Herein, we describe essential components of the study protocol to evaluate the efficacy, implementation, and cost-effectiveness of the CATSystem intervention in Kenya.

## Materials and methods

**Fig 1:**
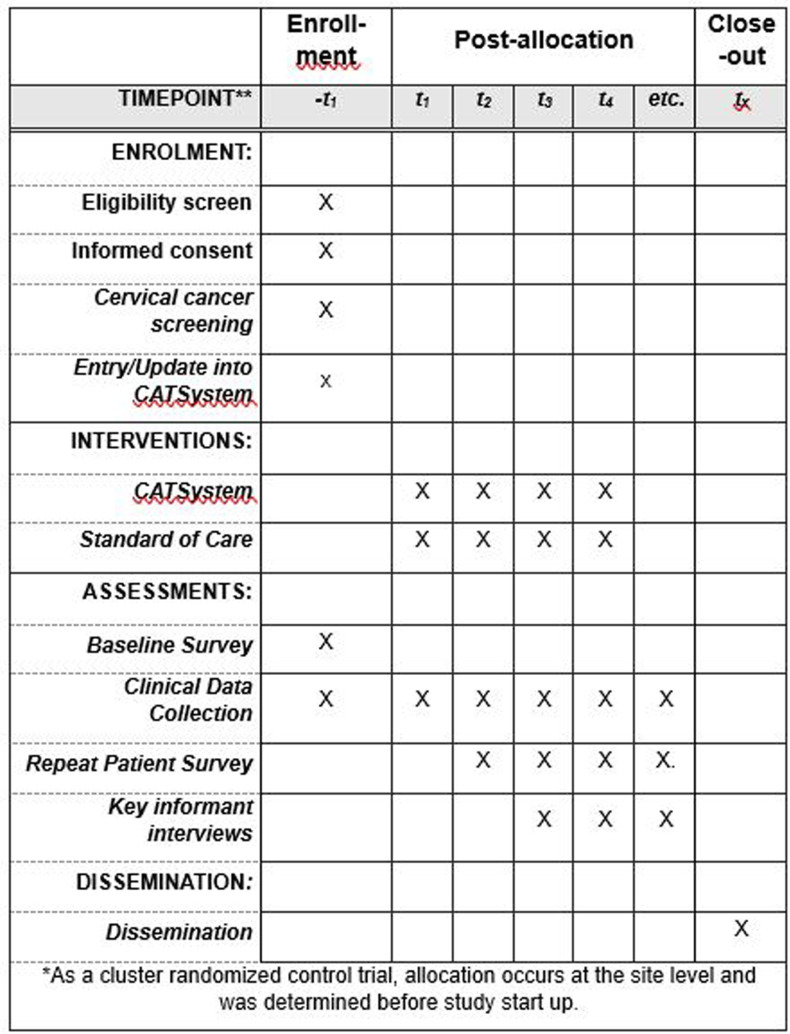
Spirit figure.

We will evaluate the CATSystem with a matched, cluster randomized controlled trial in 10 Kenyan government hospitals (5 intervention, 5 standard of care (SOC)). The specific objectives of this study are to: (1) evaluate the CATSystem in a pragmatic clinical settings testing the efficacy of the system to improve screening and treatment retention among women with a positive screen, (2) use mixed methods to assess the feasibility and acceptability of CATSystem implementation among providers and women who screened positive, and (3) calculate CATSystem costs and cost-effectiveness to inform feasibility of scale-up. The overview of study procedures can be found in Fig 1.

### CATSystem intervention overview

**Fig 2:**
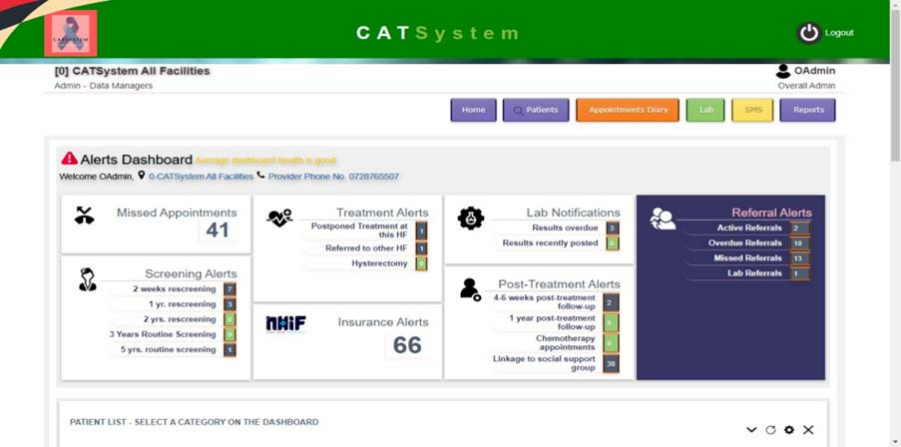
CATSystem dashboard.

The CATSystem is a systems-level eHealth intervention that aims to improve uptake and retention in the CC screening and treatment cascade of care. Primary goals of the CATSystem are to: a) increase rates of CC screening/rescreening to detect precancerous lesions or CC, and b) improve the same-day treatment, referral, and retention of women with positive screens. The CATSystem is web-based and accesses satellite broadband via modems. Its provider dashboard highlights patients with overdue services or those in need of outreach, and sends automated, customized texts to support screening and treatment follow-up per national guidelines. It also tracks the referral process, ensuring that women referred to higher level facilities present for care within a designated amount of time before prompting additional outreach and support. The CATSystem can securely store images of the cervix taken with colposcopes to allow for remote expert consultation if needed to correctly diagnose a patient. This feature extends the reach of the limited number of CC experts in the country to optimize patient care and support provider capacity and confidence with CC diagnosis. The system was designed to mirror the Ministry of Health data collection forms, is compliant with Kenya’s patient data protection laws, and can share data with Kenya’s National Cancer Registry and Demographic & Health Surveys Program helping to strengthen existing national systems. It can also be interoperable with existing EMRs in Kenya, see Fig 2.

### Site selection

Kenya has a population of over 50 million people, of whom approximately 59.4% are reproductive age girls/women (15–64 yrs). Proposed study sites will be in the Western Kenyan counties of Siaya and Busia where HIV prevalence is among the highest nationally, contributing to increased risk for cervical cancer [22,23]. Siaya has a population of 993,183 and the highest prevalence of HIV in Kenya (21%). Busia has an estimated population of 908,658, and the 5th highest HIV prevalence in Kenya at 7.7% [24]. The overall adult literacy rate is 81.5% in Kenya and 75.3% in Siaya and Busia.

### Site matching and randomization

The study sites will be matched in terms of geographic region, resource level, and patient volume resulting in five matched pairs. A random allocation process will be carried out by a study statistician, using a random number generator program, so that one site in each pair is assigned to the intervention. Study PIs and research staff will be blind to the randomization process.

### Study staffing and training

We will hire and train one full-time lay health worker as a Research Assistant (RA) per study site. Each site RA will be trained to conduct procedures specific to the study arm allocation and know the CC screening and treatment guidelines. Two Site Coordinators will oversee RAs at each site and will conduct routine site visits to provide supportive supervision to ensure adherence to study protocols, conduct data quality reviews, and provide retraining to RAs and implementing providers as needed. Site coordinators at control sites will also be tasked with entering control site clinical data to prevent unintentional intervention from site-level RAs due to enhanced data review as part of the study.

### Theoretical framework

**Fig 3:**
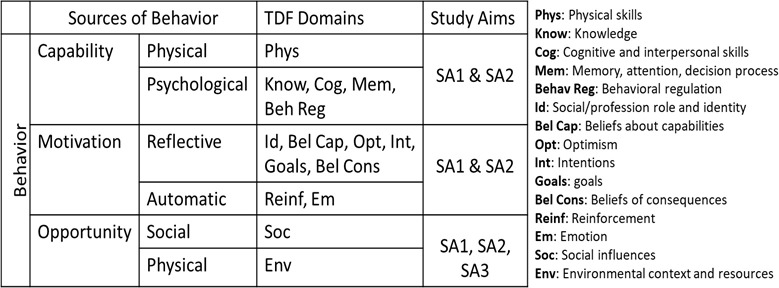
Theoretical domains framework.

This study is grounded in the Theoretical Domains Framework (TDF) (Fig 3). TDF provides a framework to examine cognitive, affective, social, and environmental/structural influences on behavior rather than propose testable relationships between elements. The central tenet of this model is that capability, motivation, and opportunity interact to influence behavior (current or desired change) [25,26]. The domains nested under capability, motivation, and opportunity include a wide range of influences: individual-level factors, such as knowledge and skills (e.g., knowledge of early detection services), social factors (social support), and environment and resource factors (e.g., access to/cost of CC treatment) [27]. For this study we will use the TDF framework to understand user priorities and needs as they use the system, then elicit feedback to assess barriers and if they are engaging with the technology as intended. We have designed a draft interview and focus group guides and surveys aligned with the TDF domains to collect data related to the intervention impact (aim 1) the acceptability of intervention implementation (aim 2), and the costs and feasibility of scaling up the CATSystem intervention (aim 3).

### Participant eligibility, consent, and ethical approval

Participant eligibility will be restricted to women >16 years of age with access to a cell phone and ability to read SMS at a 3rd-5th grade level. To maximize generalizability, all women (those living with and without HIV) receiving CC screening at the CCC or MCH/FP departments during the study period will be eligible for enrollment. Pregnant women (>20 weeks), incarcerated patients or women with impaired mental capacity affecting ability for informed consent will be excluded from the study. Women opting out of the study will be offered standard CC screening services without CATSystem enrollment. All participants will be informed of the purpose of the research, including potential benefits and risks, prior to a request for written informed consent. Research assistants familiar with CC screening procedures or healthcare providers will document study enrollment and enrollment refusal at all sites. All study methods were approved by the institutional review boards at the University of Kansas Medical Center and Jaramogi Oginga Odinga Teaching & Referral Hospital.

### Procedures

#### Sensitization meetings.

Study staff will conduct sensitization meetings at each site with personnel from all relevant departments. In these meetings, we will review the current CC screening guidelines and the national goal of 70% coverage. We will discuss the existing barriers to complete and efficient CC screening, treatment referral and follow-up, the expected advantages, and challenges of CATSystem implementation, objectives of the research, and an overview of patient engagement procedures to be followed.

**Participant surveys:** A brief enrollment survey will collect patient-level data to better understand barriers experienced by patients, and assess CC knowledge, motivation to get screened, extent of partner support, mental health, risk of violence, language preferences, and education to gain a sense of literacy levels. These instruments will assess changes that may mediate or moderate intervention impact. Women who screened positive for cancerous or precancerous lesions during the study period will be asked to complete a positive screen survey including the same measures in addition to questions assessing their CC screening experience and treatment decisions within 6 months of the positive screen or at the conclusion of treatment. Women diagnosed with invasive CC will be asked to engage in an interview to understand experiences accessing and receiving treatment at referral sites, the impact of the treatment process/disease on their psychological, social, physical, sexual, and financial health, and the type of support received and needed. Participants will complete informed consent prior to the survey and be renumerated 200 Kenyan shillings (~USD $2).

**Facility assessment form:** will be completed at all sites at the beginning, mid-point and end of study implementation to assess resource level, annual CC screening volume, and CC screening provider-patient ratios to evaluate factors that contribute to implementation feasibility overtime.

**Provider surveys:** At baseline and at the end of the study, providers in both arms will be asked to complete a survey. The survey will assess provider role/department, level of experience, knowledge and motivation regarding current CC screening and treatment guidelines, and barriers and facilitators to provision of complete CC screening services. End of study surveys will also include level of direct engagement with CATSystem and adapted quantitative items from the Acceptability of Intervention Measure (AIM), Intervention Appropriateness Measure (IAM), and Feasibility of Intervention Measure (FIM) four-item measures of implementation outcomes that are often considered “leading indicators” of implementation success [28]. Providers will be eligible to complete the survey if they (1) work primarily in CCC, laboratory, or MCH/FP departments, (2) will/have been involved in the provision of CC screening and treatment during the course of the study, and (3) have interacted with the CATSystem during the course of the study (intervention sites only). We estimate n = 5 providers per site for a total of n = 50 provider surveys (n = 25 intervention, n = 25 control). Providers will complete informed consent prior to surveys and remunerated 200 Kenyan Shillings (~USD $2).

**Supportive supervision visits:** These will occur monthly at all sites. Site Coordinators will provide technical assistance and retraining, review study materials (informed consent forms, surveys), and review data for completeness and accuracy. The monthly frequency of site visits has been adequate in previous HITSystem studies [29].

### Data collection

#### Data collection and security.

At all intervention sites, patient-specific data including demographics; phone number and patient tracing information; patient health history; dates screened and treated; lab processing of PAP and biopsy tests, lab results, and follow-up screens will be entered directly into CATSystem by team members on password secured computers. The CATSystem was built in compliance with Kenya’s 2019 Data Protection Act guidelines, is password protected, and maintains data for each client using numeric IDs to protect identities of participants and data exports in Excel. The system has been rigorously vetted by the KUMC Office of Information Security for vulnerabilities and passed both Security Headers and SSL labs test with a score of A and A + respectively. At SOC sites, all clinical and CC screening service-related data will be collected in existing paper-based registries by health care providers per routine services. To prevent unintended intervention from health workers at control sites (reviewing clinical registers and entering participant data more comprehensively than they would in SOC setting), site coordinators will review paper registries at each control site visit and electronically enter patients’ CC screening and treatment uptake data, using multiple sources (MCH, CCC, lab) to triangulate data. A parallel ‘look-alike CATSystem’ with all alerts, SMS, and patient tracking algorithms turned off, will be used for SOC data collection, to facilitate accurate, complete, and comparable data across arms. This process has been effectively used in our previous and current studies [30].

### Specific aim 1: Outcomes and analyses

**Fig 4:**
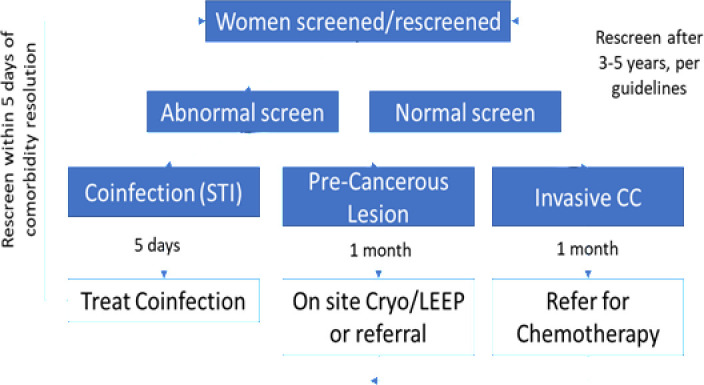
Guideline-adherent treatment algorithms.

The primary goal of SA 1 will be receipt of appropriate treatment (onsite or referral, as indicated) for clients with a positive screen. Fig 4 illustrates treatment scenarios for women with a positive screen and the targeted timeframe for treatment. Women with coinfections (cervicitis/STI) must receive treatment, resolution, and rescreening within 3 months to determine infection status and appropriate action. Patients with a positive screen for precancerous lesions should ideally receive same-day onsite treatment with cryotherapy (max duration of 5 days/1wk.) or referral (in/outside facility) to LEEP for advanced lesions (w/in 5 days/1wk.) with treatment follow-up and negative rescreen. If suspected of invasive CC, a referral to KNH or MTRH for chemo/radiotherapy within the month.

We will identify women presenting with precancerous lesions (mild, moderate or severe), suspicious of invasive CC, or those with unreliable screen results due to cervicitis/STI or another comorbidity in each arm. We will then determine if guideline adherent treatment was provided based on severity of screening results. Retention time from first screening to last follow-up will also be calculated and compared by arm. Patient and facility-level factors, including aggregated provider characteristics, will be assessed for their moderating impact on receipt of appropriate treatment after a positive screen. To assess pathways through which the CATSystem works, we will measure provider and patient information, knowledge and motivation as potential mediators pre- and post-intervention across arms.

### Statistical analyses

To demonstrate the efficacy of CATSystem versus SOC to increase the odds of women with a positive screen receiving treatment, we modeled odds of treatment as a function of study arm and selected covariates (e.g., age, partner support, HIV status) using a logistic mixed model with a random site intercept included to adjust for clustering. Between-within degrees of freedom will be specified to adjust for downward bias in standard error estimates when the number of clusters is small [31,32]. Matching of sites prior to randomization improved the balance between arms on site-level characteristics. The inclusion of covariates in the model provides additional control for between-arm differences. We will use propensity score weighting to adjust for any severe, unanticipated imbalance between arms.

In post hoc analyses we will explore baseline patient and facility-level characteristics as potential correlates and moderators of the effect of CATSystem. Effects of these characteristics will be assessed by adding main effect and study arm characteristic interaction terms to the logistic mixed model described above. We will also examine changes from baseline for key individual-level factors [HIV status, partner support (see patient baseline survey in appendix)] for the two study arms. In further analyses, we will examine provider [level of experience, knowledge and motivation] as a mediator of the intervention’s effect on odds of receiving treatment by fitting two models for each domain score: (1) A generalized or linear mixed model with change in knowledge from baseline to end of study as outcome and study arm as explanatory variable, and (2) a log-binomial mixed model with receipt of treatment as (binary) outcome and change in provider knowledge from baseline to end of study as explanatory variable. Estimates from these models will be combined to assess direct and indirect effects of the intervention [33]. We will similarly assess provider motivation, participant knowledge, and participant motivation as potential mediators.

### Specific aim 2 outcomes and analyses

The goal of SA2 is to assess the feasibility and acceptability of CATSystem implementation in government hospitals. We will use the TDF framework and elements of human centered design to develop quantitative and qualitative questions that elicit feedback from intervention users (providers and patients) to gain a better understanding of contextual factors that may act as barriers and/or facilitators to system use, and to assess CATSystem feasibility and acceptability. This feedback will be iteratively incorporated into system adjustments. We will conduct brief surveys followed by focus group discussions (FGD) with providers interacting with the CATSystem to assess barriers and facilitators to: 1) provider implementation of CATSystem, 2) patient engagement with the system, and we will 3) identify strategies for optimal CATSystem implementation given provider capacity, existing systems, and patient preferences from a strengths-based perspective. After implementation begins, we will also conduct surveys and focus group discussions (FGD) with women enrolled in the CATSystem who had a positive screen to assess users experience from the patient perspective.

#### Surveys and focus groups with providers.

We will conduct semi-annual focus group discussions during years 2 and 3 at each intervention site with all providers who routinely participate in CC screen and treatment services, including providers in the MCH/FP and CCC departments and laboratory staff. We expect 5–8 providers with diverse levels of skill, years of experience and levels of knowledge involved in CC services will participate in FGDs. The FGDs will be guided by TDF domains to understand *how* to best achieve the targeted CATSystem outcomes given the patient flow in their department and system-level challenges for the specific type of care they deliver. We will also monitor and elicit provider feedback on system and implementation modifications to better complement hospital workflow. Sample questions include: “How well does the CATSystem fit into existing work processes and practices in your setting?” (TDF: Capability, Motivation & Opportunity), “How well does CATSystem respond to the needs of your patients?” (TDF: Capability, Motivation & Opportunity) “What are your goals in the next 3 months to improve CATSystem implementation and utilization?” (TDF: Motivation). These data will enrich the quantitative survey data on acceptability and feasibility*.*

#### FGDs with women enrolled in CATSystem.

In year 3, two surveys and FGDs will be conducted at each intervention site: a) one with women enrolled in the CATSystem who received a positive screen and received appropriate treatment, and b) one with women enrolled in the CATSystem who received a positive screen and did not receive appropriate treatment. We expect 5–8 women in each of 2 FGDs per site (50–80 women total). Women who disengaged from CC care for > 6 months but re-engaged after being prompted by Aim 2 engagement will still be counted as loss to follow-up for Aim 1 analyses. Like provider FGDs, we will use TDF domains to understand how participants experienced CC care, their experience with CATSystem, and factors that motivated them to complete treatment or describe barriers to treatment.

#### Procedures.

Providers and participants who agree to participate will sign an informed consent specific to FGD participation. FGDs will be conducted in a conference room at the facility, in the language the group feels most comfortable with (English, Kiswahili, Luo, Teso, Luhya or a combination of languages) and will be audio-recorded for later transcription and translation. FGDs will last approximately 45–60 minutes, allowing opportunity to learn from each other’s experiences interacting with the CATSystem and identify opportunities to optimize the system. For their time, participants and providers of FGDs will be renumerated KSH 500 ( ≈ $2) for their time.

#### Analyses for focus group discussions.

FGDs will be digitally recorded, and notes taken with participants’ permission. Audio files will be translated and transcribed, coded, and analyzed using Dedoose, a qualitative data analysis software program. Transcriptions will be “open-coded”: identifying key words and themes driven by the 14 specific TDF domains nested within the categories of Capability, Motivation, and Opportunity and provider recommendations for optimal implementation within existing health services. We will develop a codebook with typical exemplars for each theme, calculating the frequency and distribution of themes within larger topic areas. Two coders will establish inter-rater reliability and a third coder to settle discrepancies. Demographic and individual data will be summarized with descriptive statistics.

### Specific aim 3 outcomes and analyses

The goal of SA 3 is to calculate the cost-effectiveness of CATSystem to improve quality-adjusted life-years gained. We will assess the cost-effectiveness of the CATSystem by evaluating how it impacts overall health system and societal costs and health outcomes as measured by Quality-Adjusted Life-Years (QALYs). We will adhere to the Panel on Cost-Effectiveness guidelines in Health & Medicine [34] and the ISPOR-SMDM Modeling Practices [35]. We will track resource utilization during the trial (Yrs. 2 and 3) and break out cost of the CATSystem intervention as well as follow-on screening and treatment costs in both the intervention and SOC arms. We will track screening, detection, and treatment rates from the trial. We anticipate the CATSystem will lead to higher screening costs but may lead to lower cancer treatment costs (due to treatment of earlier stages being less expensive), lower health burden, and increased survival. Because early treatment may lead to fewer cancers in the long term, those “short-term” outcomes will then be translated into long-term outcomes using a mathematical model of cervical cancer disease. Using data from the trial and from the literature, we will track productivity as well, including time (and transportation) costs to get screened, and productivity gained from effective cancer treatment.

**Fig 5:**
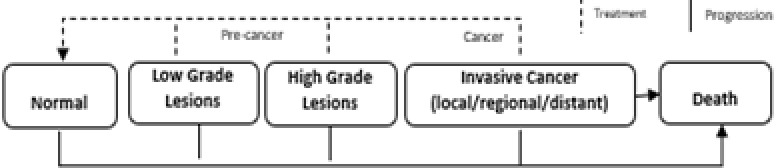
Key milestones from the Markov model of disease progression and treatment.

We will build a simplified Markov model of cancer screening, cervical cancer progression, detection, and treatment to simulate long-term health system costs and long-term length and quality-of-life under the status quo (SOC) and with the CATSystem. The model will be based on observed intervention and SOC screening and treatment rates in the trial. Our Markov model is based upon a widely-used model of cervical cancer screening interventions in developing countries [36] and is based upon a recently-published version that has been parameterized based on costs and epidemiology for Kenya [37]. Parameters from our model on disease progression, mortality, and survival (with and without treatment) will be based on the epidemiological literature relevant to Kenya. We will update costing parameters based on data collect during the trial and recent data from the local Kenyan health system partners. Key milestones from the Markov model of disease progression and treatment impact are illustrated in Fig 5.

The final outcomes of the model will be numbers of individuals screened, treated, invasive cancers, life-years, QALYs, 5-year survival, and deaths. We will calculate overall costs and QALYs for the CATSystem intervention and SOC and calculate an incremental cost-effectiveness ratio of the CATSystem compared to the SOC. We will conduct sensitivity analyses on all parameters including probabilistic sensitivity analysis (with bootstrapping of trial data and Monte Carlo simulation of other parameters) to create a posterior probability of cost-effectiveness based on uncertainty in all parameter estimates. If the system is found not to be highly cost-effective (<1x per-capita GDP/QALY), we will conduct threshold analyses to determine at what level of system cost and effectiveness it might be cost-effective.

### Power and sample size considerations

Simulations were carried out to ensure sufficient power to detect a difference in odds of guideline adherent care between the intervention and control arms. Assuming average success (treatment completion) rates of 0.10 (SOC arm) and 0.35 (CATS arm), we used R to simulate 5,000 data sets, each with outcome data for five 30-person clusters per arm. A target ICC value for each data set was randomly drawn from a half-normal distribution fit by Turner et al. (2001) [38] to the empirical distribution of 70 ICCs from studies of health outcomes with data clustered within practices, towns, or postal codes. For simulation, each target ICC was converted to a random site intercept variance on the logistic scale using the assumed SOC success rate (0.10) and the Taylor expansion approximation from Turner et al. We simulated success counts for each cluster by drawing a random cluster intercept from a Gaussian distribution with the specified variance, adding the appropriate fixed intercept and (for CATS sites) arm effect on the logistic scale, converting the resulting logit to a probability (p), and taking a random Binomial (30, p) draw. This process reflects our uncertainty in the true ICC. It also produces ICCs larger than the target values because of bias in the Taylor approximation and our use of the SOC success rate to estimate the random intercept variance, which yields a larger ICC among CATS clusters due to their higher success rate. Thus, across the 5,000 data sets, mean ICC was 0.057 for SOC clusters and 0.083 for CATS clusters (vs. mean ICC 0.023 for the half-normal distribution fit by Turner et al.). For a point of comparison, analysis of pilot screening rate data for our ten study sites yielded ICC 0.012.

For each simulated data set we used SAS 9.4 to fit a logistic mixed model with a fixed arm effect and random site intercept. Between-within degrees of freedom were specified as a small-sample adjustment [31,32]. The mean length of 95% CIs for the arm effect odds ratio was 18.0; power (1-sided test, α=0.025) was 86.9%. For comparison, the R package *cluster Power* (https://CRAN.R-project.org/package=clusterPower), which has been verified against NIH’s GRT Sample Size Calculator and PASS11, yielded power = 85.9% for the SOC and CATS rates and ICC = 0.05.

### Study oversight

The study was approved and will be overseen by the Institutional Review Boards at the University of Kansas Medical Center and Jaramogi Oginga Odinga Teaching and Referral Hospital in Kisumu, Kenya. Study team members will monitor the study for adverse events and report and adverse event to the Institutional Review Boards, who will evaluate safety. Any important protocol modifications will be reported to both IRBs prior to being implemented.

### Dissemination policy

Study results describing the rates of CC screening or rescreening to detect precancerous lesions or CC, improvement on the same-day treatment, referral, and retention of women with positive screens using the CATSystem will be widely disseminated to all key stakeholders. These include hospitals, policy makers, counties, and national level cancer prevention bodies; CC programmers and researchers worldwide; and the community of caregivers of CC. To achieve these objectives, we will take the following steps: (1) each year throughout the course of the study, study registration will be updated to track progress and communicate findings; (2) results will be communicated widely at national and international conferences as well as through publication of research papers as soon as data are available and analyzed; (3) dissemination meetings within Kenya will serve as a platform for sharing research findings thus ensuring that all players involved at the local patient, clinician, county administrator, and national policy maker levels get to know about them on time; (4) where applicable, de-identified data may be put in public repositories. Some of the key dissemination plan considerations are included in the documents for informed consent to ensure participants are informed of the plan before agreeing to study participation.

### Study status

We are currently in year 2 of the study and are actively enrolling CC screening clients at Kenyan health facilities: n = 5 intervention and n = 5 matched standard of care (SOC) sites to evaluate the efficacy of the CATSystem to improve guideline adherent CC screening and treatment retention. Participant enrollment began in February of 2024 and is expected to take approximately 24 months to complete. Participant follow up will continue for an additional 12 months post-enrollment so that outcomes among women with positive cervical cancer screens can be tracked. Results of the study are expected in 2027.

## Discussion

Although preventable, CC accounts for a significant proportion of female reproductive-tract cancers in Kenya [18]. The use of web-based tracking systems and eHealth interventions have increased health access in low-resource settings [15] and improve clinical outcomes, patient follow-up and adherence, and health communication [16]. This Cancer Tracking System (CATSystem) study protocol presents a robust approach to improving cervical cancer (CC) screening, treatment, and referral processes in Kenya, addressing critical gaps in access and quality of care. The study’s focus on leveraging web-based technology, such as the CATSystem, aligns with the global push towards digital health solutions to strengthen healthcare delivery, especially in low-resource settings [39]. By implementing a cluster randomized controlled trial (RCT) across 10 government hospitals, the study aims to assess the efficacy of the CATSystem in improving adherence to CC screening guidelines and enhancing treatment initiation for women with positive screens. The study’s emphasis on matched hospital pairs, randomization, and addressing any imbalance between arms enhances the rigor of the evaluation, increasing confidence that any observed differences in outcomes can be attributed to the intervention. The use of mixed methods, including surveys and facility assessments, adds depth to the evaluation, allowing for a comprehensive understanding of the intervention’s impact on both patients and providers. Furthermore, the study’s inclusion of feasibility, acceptability, and cost-effectiveness assessments acknowledges the importance of sustainability and scalability in implementing digital health interventions.

The potential implications of this study are significant, not only for Kenya but also for other low- and middle-income countries (LMICs) facing similar challenges in CC care. If successful, the CATSystem could serve as a model for improving cancer care in resource-constrained settings, demonstrating the value of integrating digital solutions into existing healthcare systems [40–43]. This study holds the potential to contribute valuable evidence to the field of global health, guiding the future implementation of eHealth strategies to improve cancer care and reduce mortality rates globally.

## Supporting information

S1 File Study protocol. (PDF)

S2 File Spirit checklist. (DOCX)

S3 File Inclusivity in global research questionnaire (1). (DOCX)

S4 File PLOS One human subjects research checklist. (DOCX)
