# Supplementary material for: The Cancer Tracking System (CATSystem): Study protocol of a randomized control trial to evaluate a systems level intervention for cervical cancer screening, treatment, referral and follow up in Kenya
Source: PLoS One. 2025 Feb 18;20(2):e0318941. doi: 10.1371/journal.pone.0318941 (PMC11835318; doi:10.1371/journal.pone.0318941)
Supplement: S1 File — (PDF) [file pone.0318941.s001.pdf]

University of Kansas Medical Center
RESEARCH PROTOCOL INVOLVING HUMAN SUBJECTS
TEMPLATE WITH GUIDANCE

Version date: 22 March 2023

Principal Investigator: Dr. Sarah Finocchario-Kessler (University of Kansas Medical Center), Dr. Natabhona Mabachi (American Academy of Family Physicians)

Study Title: The Cancer Tracking System (CATSystem): A Randomized control trial to evaluate a systems level intervention for cervical cancer screening, treatment, referral and follow up in Kenya.

Co- Investigator(s): Dr. Kathy Goggin (CMH), Dr. Vincent Staggs (CMH), Dr. David Hutton (Univ of Michigan), May Maloba (Global Health Innovations)

I. Purpose, Background and Rationale

A. Aim and Hypotheses

Although preventable, Cervical Cancer (CC) is the leading cause of cancer deaths among women in Sub-Saharan Africa[1] with the highest incidence in East Africa.[2] In Kenya, CC contributes to over 80% of female reproductive-tract cancers,[3] yet only 16.4% of eligible women are screened for cervical cancer[4] and 31% become lost to follow-up during treatment. Innovative and feasible eHealth strategies have improved clinical outcomes and patient retention in low resource settings and could be key to improving CC screening and treatment coverage and retention in care in Kenya. **To address system level barriers to CC screening, treatment, and follow-up, our team worked with end-users (providers and patients) to develop and pilot the Cancer Tracking System (CATSystem),** an adaptation of a web-based eHealth intervention called the HIV Infant Tracking System (HITSystem).[5] Using algorithm driven alerts for providers and SMS to patients, the CATSystem is designed to support CC screening, treatment, and referrals for reproductive age women (with/without HIV) in Kenya.

Our overall objective in this proposal is to evaluate the efficacy, implementation, and cost-effectiveness of the CATSystem to improve CC screening, treatment, referral, and follow-up care in a matched, cluster randomized controlled trial in 10 Kenyan government hospitals (5 intervention, 5 standard of care [SOC]). Specific aims (SA) include:

Specific Aim 1: Implement and evaluate the efficacy of the CATSystem to improve guideline adherent CC screening, treatment, referral, and follow-up.

Hypothesis 1: Compared to standard of care, the CATSystem will result in higher rates of CC referral, treatment, and follow-up.

H2: Adjusted for pre-implementation CC screening rates, the CATSystem will result in higher screening rates compared to rates under SOC.

SA2: Assess the feasibility and acceptability of CATSystem implementation in government hospitals.

H1: CATSystem implementation in government hospitals will be deemed feasible and acceptable.

SA3: Calculate costs and cost-effectiveness of the CATSystem to improve quality-adjusted life years gained.

H1: The CATSystem will be a cost-effective intervention to increase QALY by increasing timely CC prevention and treatment to prevent more serious disease outcomes.

B. Background and Significance

Cervical Cancer (CC) is preventable; yet in 2020, an estimated 604,000 cases were diagnosed and 342,000 deaths occurred globally.[6] Over a third of these deaths occurred in Sub-Saharan Africa even

though only 14% of the world's female population live in the region.[7] In Kenya, CC accounts for 80% of female reproductive-tract cancers.[8] Around 5,250 new cases of CC are diagnosed annually, and 3,286 women die of CC every year.[9] Women living with HIV are particularly vulnerable to CC as HIV infection is associated with increased risk of human papillomavirus (HPV) infection[10,11] and greater incidence and progression of cervical intraepithelial neoplasia (CIN).[10,12] To achieve population level health gains, the WHO recommends countries reach 70% coverage with screening and treatment. Although awareness of CC screening amongst Kenyan women is high, uptake of screening and treatment remains low; only 10% of women 18-69 years of age have received effective CC screening.[13-17] Low CC screening and treatment rates are largely due to resource constraints exacerbated by disjointed programs,[13,15,4] and systemic health inequities[18,19] driven by adverse social determinants of health (SDOH)[20].
In the ideal screen and treat protocol, treatment is administered during the same clinic visit as diagnosis for women with pre-cancerous lesions to minimize patient burden, treatment delay, and loss to follow-up (LTFU). The main modality for immediate treatment (lesion elimination) is cryotherapy techniques, which mid-level providers can be trained to perform.[14] Cases with more advanced cervical lesions are referred to provincial or referral-level hospitals for loop electrosurgical excision procedure (LEEP). Treatments for invasive CC (chemotherapy, radiation, radical hysterectomy) are referred to the highest tier referral hospitals – Moi Teaching and Referral Hospital (MTRH) in Eldoret (western Kenya) and Kenyatta National Hospital (KNH) located in the capital Nairobi. This is a barrier to treatment for many women given the geographic distance and expense.

C. Rationale

Web-based tracking systems and eHealth interventions have increased health access in low-resource settings[5] and improve clinical outcomes, patient follow-up and adherence, and health communication.[21] Necessary eHealth infrastructure is widely available in Kenya (nearly 100% access to commercial wireless signal and 90% internet penetration)[22,23] and can be leveraged to address many of the key barriers.[21,24,25,26] Our intervention **- the Cancer Tracking System (CATSystem) -** harnesses the availability of wireless technology in Kenya to improve the provision of guideline adherent cervical cancer prevention and treatment. The CATSystem utilizes algorithim-driven alerts to <u>increase coordination between key providers</u> in various departments (Maternal and child health/Family Planning (MCH/FP), Comprehensive Care Center (CCC), laboratory) by serving as a single, linked medical record for a patient's CC care. It's technology also has the ability to extend the reach of expert providers into remote areas.

CATSystem Intervention Overview. Primary goals of the CATSystem are to: a) increase rates of CC screening/ rescreening to detect precancerous lesions or CC, and b) improve the same-day treatment, referral, and follow-up rates of women with positive screens. The CATSystem is web-based and accesses satellite broadband via modems. Its provider dashboard highlights patients with overdue services or those in need of outreach, and sends automated, customized texts to support screening and treatment follow-up per national guidelines. The CATSystem can securely store images of the cervix taken with colposcopes to allow for remote expert consultation if needed to correctly diagnose a patient or can be reviewed at clinical team meetings for input. This feature extends the reach of the limited number of CC experts in the country to optimize patient care and support provider capacity and confidence with CC diagnosis. The system was designed to mirror the ministry of health data collection forms, is compliant with Kenya's patient data protection laws, and can share data with Kenya's National Cancer Registry and Demographic & Health Surveys Program helping to strengthen existing national systems. It can also be interoperable with existing EMRs in Kenya. The CATSystem tracks and documents the following points of care:
1. Counseling, 2. Patient enrollment and information, 3. CC screening, 4. Treatment for pre-cancer,
5. Referral for treatment for invasive CC, 6. Patient tracking and follow up for women anywhere along the cascade.

II. Research Plan and Design

A. Study Objectives:

Our overall objective in this proposal is to evaluate the efficacy, implementation, and cost-effectiveness of the CATSystem to improve CC screening, treatment, referral, and follow-up care in a matched, cluster randomized controlled trial in 10 Kenyan government hospitals (5 intervention, 5 standard of care [SOC]). Specific aims (SA) include:

Specific Aim 1: Implement and evaluate the efficacy of the CATSystem to improve guideline adherent CC screening, treatment, referral, and follow-up.

Hypothesis 1: Compared to standard of care, the CATSystem will result in higher rates of CC referral, treatment, and follow-up.
H2: Adjusted for pre-implementation CC screening rates, the CATSystem will result in higher screening rates compared to rates under SOC.

SA2: Assess the feasibility and acceptability of CATSystem implementation in government hospitals.
H1: CATSystem implementation in government hospitals will be deemed feasible and acceptable.

SA3: Calculate costs and cost-effectiveness of the CATSystem to improve quality-adjusted life years gained.
H1: The CATSystem will be a cost-effective intervention to increase QALY by increasing timely CC prevention and treatment to prevent more serious disease outcomes.

B. Study Type and Design:

We propose a cluster randomized controlled trial (SA1) enrolling CC screening clients at Kenyan health facilities: n=5 intervention and n=5 matched standard of care (SOC) sites to evaluate the efficacy of the CATSystem to improve guideline-adherent CC screening, treatment, referral and follow-up. SA2 will focus on the feasibility and acceptability of CATSystem implementation, and SA3 assesses the cost-effectiveness of the CATSystem and considerations for scale-up.

Study Setting: Kenya has a population of over 50 million people, of whom approximately 59.4% are reproductive age girls/women (15-64yrs). Proposed study sites will be in the Western Kenyan counties of Siaya and Busia where HIV prevalence is among the highest nationally, contributing to increased risk for cervical cancer.[27,28] Siaya has a population of 993,183 (53% female) and the highest prevalence of HIV in Kenya (21%). Busia has an estimated population of 908,658, (52% female) and the 5th highest HIV prevalence in Kenya at 7.7%.[29] The overall adult literacy rate is 81.5% in Kenya and 75.3% in Siaya and Busia. We have ensured our text messages have a 3 to 5 Flesch-Kincaid grade level. Table 1 details facility level data from the 10 most closely matched study sites from a recent retrospective review of 19 potential hospitals. Priority data points include the number of women: of reproductive age, living with HIV, screened for CC in the past year. We carefully reviewed and described the procedures and capacity for CC screening and treatment at each planned study site to ensure similarity across to optimize matching prior to randomization.

Table 1. Proposed matches from data reviewed October 2021 to September 2022										
**Site**	**Busia**	**Bondo**	**Port Victoria**	**Akala**	**Matayos**	**Ukwala**	**Yala**	**Alupe**	**Nambale**	**Khunyangu**
Repro aged women in care	51551	21368	8500	7060	5450	7196	6236	6000	5065	4156
Women in HIV care (CCC)	3306	1200	591	2018	370	663	1271	249	700	1137
Total screen/rescreen	4440	1878	1087	1003	359	477	617	478	730	615
% repro-age women	8.6%	8.8%	12.8%	14.2%	6.6%	6.6%	9.9%	8.0%	14.4%	14.8%
Trained providers (screening)	10	10	8	6	7	6	3	5	4	3

Of 19 sites reviewed, this table shows the best 10 matched sites. All sites have VIA/VILI available for screening and will provide on site treatment with cryotherapy and/or thermocoagulation. Documentation of abnormal screening was variable due to inconsistent documentation. Based on data, we estimate an abnormal screen rate of 1%-5%.

Study Design: We propose a matched, cluster randomized controlled study design [2 arms, 5 intervention and 5 control hospitals (clusters), and at least n=30 patients with a positive CC screening result per cluster] to evaluate the efficacy of the CATSystem. The methods for Aims 1-3 are described below.

Staffing and responsibilities. We will hire and train one full time lay health worker as a Research Assistant (RA) per study site. Each site RA will be trained to conduct procedures specific to the study arm allocation and know the CC screening and treatment guidelines. Lay health workers have been effective and dependable RAs in prior HITSystem studies and programmatic work as they are already embedded in the facility/community and provide an opportunity to leverage existing relationships and build capacity through a train the trainer model. At all sites, RAs will assess eligibility and conduct informed consent procedures and surveys. At intervention sites, identified clinical staff will be trained on features of the system including how to routinely enter and update patient data, generate required county reports and follow-up with patients as appropriate. RAs will do the majority of data entry in the CATSystem at each participant visit, updating clinical information (services received, lab results, comorbidities, treatment, etc.) per clinician notes. RAs will also lead patient outreach and follow-up efforts (phone or SMS via CATSystem) and coordinate physical tracking, if needed, particularly for diagnoses that require timely treatment. Two Site Coordinators experienced in coordinating research activities on the ground will divide the responsibilities of overseeing activities at the 10 study sites.

Procedures.

Data Collection and Security: At all study sites, patient-specific data including demographics; phone number and patient tracing information; patient health history; dates screened and treated; lab processing of PAP and biopsy tests, lab results, and follow-up screens will be entered directly into CATSystem by team members on password secured computers. The CATSystem was built in compliance with Kenya's 2019 Data Protection Act guidelines, is password protected, and maintains data for each client using numeric IDs to protect identities of participants and data exports in Excel. The system has been rigorously vetted by the KUMC Office of Information Security for vulnerabilities and passed both Security Headers and SSL labs test with a score of A and A+ respectively. At SOC sites, all clinical and CC screening service-related data will be collected in existing paper-based registries by health care providers per routine services. To prevent unintended intervention from health workers at control sites (reviewing clinical registers and entering participant data more comprehensively than they would in SOC setting), site coordinators will review paper registries at each control site visit and electronically enter patients' CC screening and treatment uptake data, using multiple sources (MCH, CCC, lab) to triangulate data. A parallel 'look-alike CATSystem' with all alerts, SMS, and patient tracking algorithms turned off, will be used for SOC data collection, to facilitate accurate, complete, and comparable data across arms. This process has been effectively used in our previous and current cRCT studies.

Participant surveys: A brief enrollment survey will collect patient-level data to better understand barriers experienced by patients, and assess CC knowledge, motivation to get screened, extent of partner support, mental health, risk of violence, language preferences, and education to gain a sense of literacy levels (both arms, Appendix 1). These instruments are modified from those used in HITSystem studies to assess changes that may mediate or moderate intervention impact. Women who screened positive for precancerous lesions during the study period will be asked to complete a survey including the same measures in addition to questions assessing their CC screening experience and treatment decisions within 6 months of the positive screen or at the conclusion of treatment (both arms, Appendix 2). Women diagnosed with invasive CC will be asked to engage in an interview to understand experiences accessing and receiving treatment at referral sites, the impact of the treatment process/disease on their psychological, social, physical, sexual, and financial health, and the type of support received and needed (both arms, Appendix 3). Costs associated with seeking screening and treatment will be included in these surveys. The remuneration plan for participant and provider surveys are outlined in the Human Subjects section.

Facility assessment form: will be completed at all sites at the beginning, mid-point and end of study implementation to assess resource level, annual CC screening volume, and CC screening provider-patient ratios to evaluate factors that contribute to implementation feasibility overtime. (See Appendix 4)

Provider surveys: At baseline and at the end of the study, providers in both arms will be asked to complete a survey (See Appendix 5). The survey will assess provider role/department, level of experience, knowledge and motivation regarding current CC screen and treat guidelines, and barriers and facilitators to provision of complete CC screening services. End of study surveys will also include level of direct engagement with CATSystem and adapted quantitative items from the Acceptability of Intervention Measure (AIM), Intervention Appropriateness Measure (IAM), and Feasibility of Intervention Measure (FIM) four-item measures of implementation outcomes that are often considered “leading indicators” of implementation success.[30,31] Providers will be eligible to complete the survey if they (1) work primarily in CCC, laboratory, or MCH/FP departments, (2) will/have been involved in the provision of CC screening and treatment during the course of the study, and (3) have interacted with the CATSystem during the course of the study (intervention sites only). We estimate n=5 providers per site for a total of n=50 provider surveys (n=25 intervention, n=25 control). Providers will complete informed consent prior to surveys.
Supportive Supervision Visits will occur monthly at all sites. Site Coordinators will provide technical assistance and retraining, review study materials (informed consent forms, surveys), and review data for completeness and accuracy. The monthly frequency of site visits has been adequate in previous HITSystem studies.

Aim 2 methods will include surveys and focus groups with CATSystem users, described below:
Surveys & focus groups with providers: We will conduct semi-annual surveys (yrs 2 and 3) and FGD at each intervention site with all providers who routinely participate in CC screen and treatment services, including providers in the MCH/FP and CCC departments and laboratory staff (Appendix 6). We expect 5-8 providers with diverse levels of skill, years of experience and levels of knowledge involved in CC services at each site will participate in FGDs. The FGDs at intervention sites will be guided by TDF domains to understand *how* to best achieve the targeted CATSystem outcomes given the patient flow in their department and system-level challenges for the specific type of care they deliver. We will also monitor and elicit provider feedback on system and implementation modifications to better complement hospital workflow. *To ensure we are responsive to providers we will conduct FGD check in’s twice a year in years 2 and 3 (see Appendix).*

FGDs with women enrolled in CATSystem: In year 3, two surveys and FGDs will be conducted at each intervention site: a) one with women enrolled in the CATSystem who received a positive screen and received appropriate treatment, and b) one with women enrolled in the CATSystem who received a positive screen and did not receive appropriate treatment. We expect 5-8 women in each of 2 FGDs per site (50-80 women total). Women who disengaged from CC care for >6 months but re-engaged after being prompted by Aim 2 engagement will still be counted as loss to follow-up for Aim 1 analyses. Similar to provider FGDs

we will use TDF domains to understand how participants experienced CC care, their experience with CATSystem, and factors that motivated them to complete treatment or describe barriers to treatment, see Appendix 7.

Procedures: Providers and participants who agree to participate will sign an informed consent specific to survey and FGD participation, respectively. FGDs will be conducted in a conference room at the facility, in the language the group feels most comfortable with (English, Kiswahili or both) and will be audio-recorded for later transcription and translation. FGDs will last approximately 30-45 minutes, allowing opportunity to learn from each other's experiences interacting with the CATSystem and identify opportunities to optimize the system.

Analyses for focus group discussions: FGDs will be digitally recorded and notes taken with participants' permission. Audio files will be translated and transcribed, coded, and analyzed using Dedoose, a qualitative data analysis software program. Transcriptions will be "open-coded": identifying key words and themes driven by the 14 specific TDF domains nested within the categories of Capability, Motivation, and Opportunity and provider recommendations for optimal implementation within existing health services. We will develop a codebook with typical exemplars for each theme, calculating the frequency and distribution of themes within larger topic areas. Two coders will establish inter-rater reliability and a third coder to settle discrepancies. Demographic and individual data will be summarized with descriptive statistics.

Aim 3 will use data collected in Aims 1 and 2 to calculate the cost-effectiveness of the CATSystem.

C. Sample size, statistical methods, and power calculation

Outcomes. The primary outcome of SA 1 will be receipt of appropriate treatment (onsite or referral, as indicated) for clients with a positive screen. Figure 5 illustrates treatment scenarios for women with a positive screen and the targeted timeframe for treatment. Women with coinfections (cervicitis/STI) must receive treatment, resolution, and rescreening within 3 months to determine infection status and appropriate action. Patients with a positive screen for precancerous lesions should ideally receive same day onsite treatment with cryotherapy (max duration of 5 days/1 wk) or referral (in/outside facility) to LEEP for advanced lesions (w/in 5 days/1 wk) with treatment follow-up and negative rescreen. If suspected of invasive CC, a referral to KNH or MTRH for chemo/ radiotherapy within the month.

We will identify women presenting with precancerous lesions (mild, moderate or severe), suspicious of invasive CC, or those with unreliable screen results due to cervicitis/STI or other comorbidity in each arm. We will then determine if guideline adherent treatment was provided based on severity of screening results. Retention time from first screening to last follow-up will also be calculated and compared by arm. Patient and facility-level factors, including aggregated provider characteristics, will be assessed for their moderating impact on receipt of appropriate treatment after a positive screen. To assess pathways through which the CATSystem works, we will measure provider and patient information, knowledge and motivation as potential mediators pre- and post-intervention across arms.

Secondary Outcome: We will determine whether each participant is screened for CC during the intervention period and use these data to assess the effect of the CATSystem intervention on odds of screening. To adjust for sites' pre-intervention screening rates, we will compute the proportion of women screened for CC cancer during the pre-intervention period and include this variable as a covariate in modeling.

Statistical analyses for SA1 primary outcome. To demonstrate the efficacy of CATSystem verses SOC to increase the odds of women with a positive screen receiving treatment, we will model odds of treatment as a function of study arm and selected covariates (e.g., age, partner support, HIV status) using a logistic mixed model with a random site intercept included to adjust for clustering. Between-within degrees of freedom specified to adjust for downward bias in standard error estimates when the number of clusters is not large.[32,33] Matching of sites prior to randomization will improve balance between arms on site-level

characteristics. Inclusion of covariates in the model will provide additional control for between-arm differences. We will use propensity score weighting to adjust for any severe, unanticipated imbalance between arms.

In post hoc analyses we will explore baseline patient and facility-level characteristics as potential correlates and moderators of the effect of CATSystem. Effects of these characteristics will be assessed by adding main effect and study arm×characteristic interaction terms to the logistic mixed model described above. We will also examine changes from baseline for key individual level factors [HIV status, partner support (see patient baseline survey in appendix)] for the two study arms. In further analyses, we will examine provider [level of experience, knowledge and motivation (see Provider survey in appendix)] as a mediator of the intervention's effect on odds of receiving treatment by fitting two models for each domain score: (1) A generalized or linear mixed model with change in knowledge from baseline to end of study as outcome and study arm as explanatory variable, and (2) a log-binomial mixed model with receipt of treatment as (binary) outcome and change in provider knowledge from baseline to end of study as explanatory variable. Estimates from these models will be combined to assess direct and indirect effects of the intervention.[34] We will similarly assess provider motivation, participant knowledge, and participant motivation as potential mediators.

Sample size considerations. Assuming average success (treatment completion) rates of 0.10 (SOC arm) and 0.35 (CATS arm), we used R to simulate 5,000 data sets, each with outcome data for five 30-person clusters per arm. A target ICC value for each data set was randomly drawn from a half-normal distribution fit by Turner et al. (2001)[35] to the empirical distribution of 70 ICCs from studies of health outcomes with data clustered within practices, towns, or postal codes. For simulation, each target ICC was converted to a random site intercept variance on the logistic scale using the assumed SOC success rate (0.10) and the Taylor expansion approximation from Turner et al. We simulated success counts for each cluster by drawing a random cluster intercept from a Gaussian distribution with the specified variance, adding the appropriate fixed intercept and (for CATS sites) arm effect on the logistic scale, converting the resulting logit to a probability (p), and taking a random Binomial(30, p) draw. This process reflects our uncertainty in the true ICC. It also produces ICCs larger than the target values because of bias in the Taylor approximation and our use of the SOC success rate to estimate the random intercept variance, which yields a larger ICC among CATS clusters due to their higher success rate. Thus, across the 5,000 data sets, mean ICC was 0.057 for SOC clusters and 0.083 for CATS clusters (vs. mean ICC 0.023 for the half-normal distribution fit by Turner et al.). For a point of comparison, analysis of pilot screening rate data for our ten study sites yielded ICC 0.012.

For each simulated data set we used SAS 9.4 to fit a logistic mixed model with a fixed arm effect and random site intercept. Between-within degrees of freedom were specified as a small-sample adjustment.[32,33] The mean length of 95% CIs for the arm effect odds ratio was 18.0; power (1-sided test, α=0.025) was 86.9%. For comparison, the R package *clusterPower* (https://CRAN.R-project.org/package=clusterPower), which has been verified against NIH's GRT Sample Size Calculator and PASS11, yielded power=85.9% for the SOC and CATS rates and ICC=0.05.

The proportion of screened women with a positive result ranged between 3.4% (53/1576) during the SOC period and 7.1% (39/550) during the intervention period. **To guide enrollment targets, we will assume a positive screen rate of 5%, which would require enrollment of at least n=600 women per cluster to result in 30 women with a positive screen. We will enroll an additional 10% to account for attrition due to relocation, transfer of health facility or death.** During our pilot study, we enrolled 552 patients at the pilot facility over the span of 15 months, however, several months were impacted by Covid-19 restrictions at the health facility (n=3) or a clinical worker strike (n=1) when enrollment numbers dropped from an average of 53 to 24 patients per month. We thus estimate the potential to have enrolled up to 795 patients under more normal conditions. We also anticipate that monthly enrollment numbers may decrease over the 24-month period, given that once a woman has been screened and/or treated, their next screening appointment per guidelines would be within 3 to 5 years; thus fewer women at the health facility will be eligible for screening over the course of the study. With this in mind, **we expect a 24 month enrollment**

period will be adequate to reach a total of 660 participants at each hospital (660 x 10 sites= 6,600 study participants).

Missing data; multiple testing. Taking an intent-to-treat approach, rather than exclude participants who drop out from analyses we will carry out and compare two analyses, one where dropouts are classified as not receiving treatment, and another where inverse probability of attrition weighting is used to adjust for participants lost to attrition.[36] Missing data on explanatory variables will be imputed using stochastic regression imputation or, if the rate of missing data exceeds 1%, using multiple imputation by chained equations. Consistent with guidance from the statistics community,[37,38] we will focus on effect sizes and their confidence intervals rather than treating $p<0.05$ as a dichotomous indicator of whether an effect is practically important and classifying results as “significant” or “not significant” on this basis.[39] Model selection processes that exploit chance will be avoided.

Analysis plan for secondary outcome of CC screening. As for the primary outcome, we will assess the CATSystem effect for the secondary outcome by modeling odds of CC screening as a function of study arm using a logistic mixed model with a random site intercept, specifying between-within degrees of freedom. In addition, we will adjust for sites’ pre-intervention screening rate by including this variable as a covariate. This is similar to computing a change score for each site and assessing the between-arm “difference in differences” but (1) allows modeling data at the patient level and examination of patient-level characteristics as correlates of outcomes, and (2) involves estimation of a regression coefficient reflecting association between sites’ pre- and post-implementation screening rates, thereby avoiding assumption of perfect reliability implicit in the change score approach. We used the R package *clusterPower* to estimate statistical power for this outcome, assuming an SOC screening rate of 15% (an improvement from the 10.5% rate observed in our pilot data), a CATSystem screening rate of 30%, and an ICC of 0.04, four times the ICC observed for the ten study sites’ screening rates in our pilot data. The proposed sample size (600 study completers per site) yields 82.5% power for a 1-sided tests at $\alpha=0.05$. This does not account for reduction in error variance we will gain by including sites’ pre-intervention screening rates as a covariate in modeling.

D. Subject Criteria (See Vulnerable Populations appendix, if applicable):

Health Facility Eligibility and Selection. Eligible sites must provide CCC and MCH/FP services and serve an average at least 4000 reproductive aged women. A repeated assessment of the previous three months will be conducted prior to study start to capture any changes that may require adjustments to the planned sites. All eligible facilities will be yoked in pairs matched on volume of reproductive aged women, average number of women screened per month, and screen and treat capacity. The study statistician will randomly allocate one site in each pair to the intervention.

Participant Eligibility and Consent. Eligibility will be restricted to women >16 years of age (age of treatment consent is 16 yrs) with access to a cell phone and ability to read SMS’s at a 3rd-5th grade level. While literacy levels in Bungoma and Busia are high, women can opt to customize SMS messages to include pre-specified familiar greetings or numbers to indicate the need to contact or return to the health facility (e.g. ‘afya’ [Swahili for healthy] or ‘7777’). To maximize generalizability, all women receiving CC screening at CCC or MCH/FP during the study period will be eligible for enrollment. Pregnant women (>20 weeks) who are not eligible for CC screening per Kenyan guidelines, incarcerated patients or women with impaired mental capacity affecting ability for informed consent will be excluded from the study. Women opting out of the study will be offered standard CC screening services without CATSystem enrollment. All participants will be informed of the purpose of the research, including potential benefits and risks, prior to a request for written informed consent. Guardians of eligible girls <18 years of age will be required to sign a written, informed consent and the eligible girl will be asked to provide written assent. Site Research Assistants (trained lay health workers) familiar with CC screening procedures and providers will document study enrollment and enrollment refusal at all sites.

E. Specific methods and techniques used throughout the study

All laboratory tests (i.e. VIA/VILI, HPV testing, PAP for cervical cancer screening) and clinical management (i.e. Cryotherapy, thermocoagulation, LEEP, chemotherapy, radiation for cervical cancer treatment) will be provided by trained clinical providers at the study hospitals, per national guidelines and standard of care procedures. That CATSystem will prompt and support patient retention through these routine clinical services but will not provide additional laboratory diagnostic tests or treatments outside of the standard of care recommendations based on the participant's clinical presentation. All samples will be handled, stored, and destroyed per the hospital's established procedures.

F. Risk/benefit assessment:

1. **Physical risk**: While providers are most experienced with cervical cancer screening and treatment methods, women receiving screening and/or treatment may experience minimal pain and discomfort associated with standard screening and treatment procedures. While this isn't a risk unique to study participation, study staff will work with clinical staff to ensure women are informed about and prepared for the screening and treatment process. Participants in the intervention arm and in the control arm are at equal risk of experiencing this risk. Women will be counseled through the process of screening and treatment, including be given information about what to expect in terms of discomfort. Training on procedures will be reinforced to minimize risk of errors and need for repeat screenings.
2. **Psychological risk**: The risk of psychological and emotional distress exists, particularly for women receiving a positive screen. While this isn't a risk unique to study participation, if this occurs, we will provide assistance through study staff, clinic peer counselors and - as necessary - clinic medical staff. The risk of psychological distress exists equally among women in the intervention and control arms.
3. **Social risk**: Loss of confidentiality and stigma are concerns. In a setting in which a woman may not have disclosed her cervical cancer screening status to her partner, inadvertent disclosure is a concern. Participants could be treated unfairly or discriminated against, or could face problems being accepted by their partner, family or community. This is a greater concern at intervention sites using the CATSystem, as the CATSystem will send SMS to women to prompt return to the hospital. To protect confidentiality, the following steps will be taken: Study staff will be trained regarding the need to strictly protect confidentiality; this will also be reinforced with the clinic staff. Prior to starting the study, we will meet with clinic staff and peer counselors to identify any additional strategies that could be taken in that particular setting to help improve participant confidentiality. This includes strict adherence to protocols for the content of CATSystem text messages and procedures for those engaged in patient tracing in the community. Messages sent by the CATSystem were designed to protect patient confidentiality, by not referring to cervical cancer. Loss of confidentiality is also a risk for participants of the focus group discussions and surveys. While facilitators will emphasize the need for confidentiality amongst participants, confidentiality cannot be fully guaranteed in a focus group setting. This will be explained to participants.
4. **Economic risk**: The CATSystem will prompt patient retention in standard of care clinical services; however, will not financially support the provision of these services. In instances where women are prompted to return for services by the CATSystem – but would not have otherwise returned – they will be responsible for covering the costs of the services they receive.
5. **Potential benefit of participating in the study**: The potential for individual benefit from study participation exists, particularly among women enrolled in the intervention arm. In preliminary data, the CATSystem improved retention in cervical cancer screening and treatment. Early detection of

cervical cancer and early treatment can significantly improve prognosis and reduce costs later. Findings from this study will make an important contribution to cervical cancer services in Kenya and other comparable low resource countries and will contribute to the limited data regarding the implementation of eHealth strategies in this context. As Kenyan and global recommendations move toward eHealth and screen and treat strategies, it is critical to assess the performance and cost-effectiveness of these methods in resource-constrained settings. This data will provide evidence for Ministries of Health and other organizations to consider optimal mHealth strategies to meet the global cervical cancer goals.

G. Location where study will be performed:

The informed consent interview and enrollment survey will be conduct at the participant's hospital of enrollment, in a private room. CATSystem data entry at enrollment and at all subsequent participant encounters will also be conducted at the hospital of the participant's enrollment. Cervical cancer screening and most treatments (thermocoagulation, cryotherapy) will be conducted at the participants hospital of enrollment. In instances where participants need more intensive treatment (chemotherapy, radiation) they may be referred to a higher level facility, where these services will be performed and documented within the CATSystem.

Paper-based records (informed consent, paper surveys) will be stored in a locked filing cabinet at the study hospital. They will be collected by Co-I Maloba on a quarterly basis and then be stored at the Global Health Innovations office in Nairobi until study completion.

H. Collaboration (with another institution, if applicable): This is a collaborative effort between the University of Kansas Medical Center (KUMC), American Academy of Family Physicians (AAFP), Children's Mercy Hospital (CMH), University of Michigan (UMich), and Global Health Innovations Kenya (GHI). KUMC will serve as the lead IRB for the study and AAFP, CMH, and UMich will rely on their approval. Study approval will also be sought by GHI through the Kenya Medical Research Institute. As the in-country approving IRB, consent forms will be drafted using their template.

I. Single IRB Review for a Multi-site study (if applicable):

1. **For which sites will KUMC serve as the IRB of record?** KUMC, AAFP, CMH, UMich
2. **Indicate which study activities will occur at each site. If all study procedures will be identical across study sites, state this.** The table below outlines the sites participating in this study and their roles in study implementation

	Organization	Study Role
**Administrative Sites**	University of Kansas Medical Center	Annual Conflict of Interest and Human Subjects Research Certification for all KUMC and GHI study members; study design, implementation and management, data analysis and dissemination
	Kenya Medical Research Institute	Institutional Review Board and adverse event monitoring; liaison with hospital and government administrations in study regions; long term storage of study records
	Children's Mercy Hospital; Kansas	Support for study design, implementation, and data analysis

	City, MO (CMH)	
	University of Michigan	Support for study design, implementation, and data analysis
**Data Coordinating Sites**	Global Health Innovations (GHI)	CATSystem technical support, oversight of Study Coordinators monitoring daily study activities (informed consent, data collection, patient follow-up) at all study sites
**Enrollment/Participating Sties**	10 Kenyan health facilities	CATSystem and study enrollment, HIV care service provision, onsite VL sample collection, temporary storage of study records

3. **Describe how you will assess the capacity of each site to perform the research (e.g., expertise, staffing, space, equipment, etc.) If applicable, include site evaluation tools in your IRB submission**. We will hire and train one full time research assistant for each study hospital. Each RA will be trained to conduct study procedures specific to the study arm allocation. RAs have demonstrated effective and reliable HITSystem use in our prior studies and programmatic work. At all sites, RAs will assess eligibility, conduct informed consent procedures and surveys, and facilitate participant follow up. At sites implementing CATSystem, RAs will be the primary persons to enter and routinely update patient data. RAs also will lead patient outreach and follow-up efforts (phone or texting via CATSystem). Site Coordinators will routinely visit each study hospital to provide supportive supervision to ensure adherence to study protocols.

4. **Describe how the lead investigators will ensure that all participating sites use the IRB-approved version of the protocol, consent, recruitment materials and other study documents**. Hard and soft copies of all study related documents will be provided to participating sites. If changes are made to any of the materials, the newer versions will be provided as soon as IRB approval is received. At each visit, site coordinators will ensure that the most recent version of documents are being used. At site visits, site coordinators will also collect the informed consents from the site to give to Co-I Maloba for storage. Maloba will review all study documents upon receipt to ensure that the appropriate version is being used and that the forms are being filled out correctly. If she has any concerns, she will work with the site and the site coordinator to schedule a follow up training. Since data collection occurs through the web-based CATSystem, all changes will be automatically and immediately applied across study sites, ensuring that the most recent version of the tool is being used by all sites.

5. **Describe how the lead investigators will communicate with and disseminate new information to other sites (e.g., training meetings, regularly-scheduled conference calls, notifications, etc**.) The lead investigator will communicate with each of the sites in the following ways:
    - Prior to study launch, a startup training will provided to all study sites. This training will cover purpose of the study, conducting informed consent, enrolling participants, and using the site's designated point of care testing system.
    - Twice monthly calls between the US-based team, Kenya-based Co-Is, and the site coordinators assigned to each study site will allow the lead investigators to check up on study progress and communicate new information to site coordinators.

- Site coordinators will make monthly visits to study sites to check up on study progress and disseminate any relevant information to clinical staff. Additional phone or email follow up between study sites and site coordinators will occur to address issues that come up.
- Email or additional calls between US-based personnel, Kenya-based personnel, and study sites will occur, as necessary.

6. **Describe how the lead investigator will assess protocol compliance, unanticipated problems and adverse events at other sites**. US-based study staff will review study-related material (including informed consents and data collected through the CATSystem) at least quarterly. Any concerns with the consent forms or the data collection will be immediately addressed. Monthly calls between US and Kenya-based research staff will allow site coordinators to check in with Finocchario Kessler after each site visit to discuss study progress and concerns. Maloba will make quarterly visits to all study sites to assess protocol compliance and address any issues and adverse events. Staff at each study site will have Maloba's and their designated site coordinator's phone numbers and email addresses, allowing them to contact research personnel if any questions or issues arise. A designated field in the CATSystem and control site data sheets allows clinical and research staff to document any adverse events.

7. **Name the member of the KUMC study team who will be the point of contact to coordinate oversight and communication with the sites**. PI Finocchario-Kesser will be the point of contact to coordinate oversight and communication with the sites.

J. Community-Based Participatory Research (if applicable)
N/A

K. Personnel who will conduct the study, including:

1. Indicate, by title, who will be present during study procedure(s): Clinical staff, research assistants, and/or site coordinator
2. Primary responsibility for the following activities, for example:
   a. Determining eligibility: Research assistant
   b. Obtaining informed consent: Research assistant
   c. Providing on-going information to the study sponsor and the IRB: PIs Finocchario-Kessler and Mabachi
   d. Maintaining participant's research records: Research assistant, site coordinator, Co-I Maloba
   e. Completing physical examination: Clinical staff
   f. Taking vital signs, height, weight: Clinical staff
   g. Drawing / collecting laboratory specimens: Clinical staff
   h. Performing / conducting tests, procedures, interventions, questionnaires: Clinical staff or Research Assistant
   i. Completing study data forms: Research assistant and/or study coordinator
   j. Managing study database: Study Statistician

L. Assessment of Subject Safety and Development of a Data and Safety Monitoring Plan

An external DSMB will be established to review and evaluate the accumulated study data for participant safety, study conduct, and progress on an annual basis. The DSMB membership will include a minimum of three members: one biostatistician, one epidemiologist, and one or more clinician with expertise in obstetrics, gynecology, oncology and cervical cancer care in Kenya. All members will have expertise in the conduct and methodology of clinical trials. Given geographic distances, DSMB communication will be conducted via email and phone, with annual meetings conducted using web-based conferencing. All serious adverse events will be reported to IRBs in both countries and to the DSMB. Based on these assessments, the DSMB will make recommendations regarding the continuation, modification or early termination of the study. The DSMB will establish early stopping protocols prior to study initiation.

III. Subject Participation

A. **Recruitment**: All women who receive cervical cancer screening at study hospitals will be offered enrollment into the study. Clinical staff or the research assistant may identify eligible women from the Maternal and Child Health/Family Planning (MCH/FP) or comprehensive care centers (CCC). Once identified, the research assistant assigned to each site will inform eligible women about the purpose of the study, will describe risk/benefits or the study, and will describe what to expect from participation in the study before asking women if they'd like to enroll in the study. At control sites, the standard of care services will be described to participants. At intervention sites, the standard of care services and the unique communication features of the CATSystem will be described to patients. Eligible women will sign a written informed consent form in either Kiswahili or English (per the participant's preference) prior to enrollment. If a woman is non-literate, a thumbprint will be used in lieu of a written signature. Upon enrollment, the RA will conduct a brief enrollment survey with each participant. Participant will be remunerated $2.00 for participating in the enrollment survey, in appreciation of their time.

B. **Screening Interview/questionnaire**: N/A

C. **Informed consent process and timing of obtaining of consent**

All participants will be informed of the purpose of the research, including the potential benefits and risks of CC screening and treatment and, at CATSystem-implementing sites, the unique communication features of the CATSystem. The RA will go over the informed consent with each woman and then allow her to read it over on her own. RA will explain that all participants have the right to discontinue participation at any time and that women who decline study participation will access standard of care cervical cancer screening and treatment services. At CATSystem study sites, caregivers declining study participation can also choose to be tracked in the CATSystem without providing additional samples for VL tests.

Prior to a request for written informed consent, women will be asked to summarize what they've been told/read to ensure comprehension. Women will then be asked if they would like to enroll with their infants by: a) providing their cell phone contact and/or tracing map from the hospital to their residence to facilitate follow up and (b) allowing their patient information to be entered and tracked in the CATSystem (at CATSystem sites only). Women who are non-literate will have the option of using a thumb-print in lieu of a signature for study consent.

Girls between the ages of 16 and18 are eligible for inclusion in this study, with consent from a legal guardian and assent from the girl. RAs will take additional time with these young participants to

ensure comprehension, emphasizing that participation is optional and is not a requirement for receiving standard of care cervical cancer screening and treatment services.

The contact number of the Site Coordinator and in-country PI will be provided to all who agree to participate. Participants will be encouraged to call this number if they have questions about their participation in the study. IRB approval will be sought from an institution in Kenya and the University of Kansas Medical Center.

D. **Alternatives to Participation:** Women who choose not to participate in the study will receive standard cervical cancer screening and treatment at the hospital, without CATSystem tracking and/or additional study measures (e.g. enrollment survey). At intervention hospitals, women who choose not to participate in the study can still opt-in to CATSystem enrollment programmatically and choose to have their data excluded from study analyses. Women will be made aware of these alternatives during the informed consent process.

E. **Costs to Subjects:** N/A

F. **How new information will be conveyed to the study subject and how it will be documented:** Providers or the site coordinator will inform participants of new information at their next clinic appointment. Documentation of all new information or procedures will be kept in the study records. Documentation of a woman's notification of new information will be noted in the "comments" field of the woman's CATSystem entry.

G. **Payment, including a prorated plan for payment:** Upon enrollment, the RA will conduct a brief enrollment survey with each participant. Participant will be remunerated $1.00 for participating in the enrollment survey, in appreciation of their time. Participants in Aim 2 qualitative components will receive $5.00 in remuneration to support travel to/from the hospital and in appreciation of their time. Travel costs will not be reimbursed

H. **Payment for a research-related injury:** N/A

IV. Data Collection and Protection

A. Data Management and Security:

At intervention sites, all relevant clinical data at intervention sites will be captured through the CATSystem's online, automated system which produces output in Excel format. Patient specific information will be entered by the RA at the hospital during the first and subsequent consultations to capture relevant demographic and clinical data and laboratory results. Data are maintained for each woman within the password-protected CATSystem using a numeric ID to protect the identity of all participants. The site coordinators will routinely visit CATSystem sites for data quality assurance. At non-CATSystem sites, RAs will use individual patient files, augmented by available hospital paper-based registers and the national laboratory online database to collect relevant routine clinical data to enter study data into a secured electronic database. As with CATSystem data, each woman will be identified by a numeric study ID to protect their identify. RAs will be the only ones with access to the codes linking participants' identity with their study ID.

At enrollment, participants will take a brief survey assessing individual characteristics. RAs will conduct these surveys on paper forms. Site coordinators will then enter the responses from paper forms into a secured electronic database. Additional paper-based study logs kept at study sites will

document study enrollment, enrollment refusal, adverse events that occur as a result of the study, and communication between clinical or study staff and participants. Hospital or national-level events that may impact clinical care and study operations will be documented across all sites (intervention and control). Logs will be filled out by personnel performing the respective actions. The site coordinator will collect study logs and surveys routinely at all study sites and enter them into a secured electronic database.

All study staff will be trained to promote standardized and objective collection and recording of participant information. Study personnel will review data entered in the CATSystem and other study data collection tools for legibility, consistency, and completeness. All data will be stored in a password-protected database that is backed up through a secure offsite connection. All CATSystem data will be stored and backed-up on secure servers in Kansas City, USA.

B. Sample / Specimen Collection: All samples will be collected per established procedures at participating health facilities

C. Tissue Banking Considerations: N/A

D. Procedures to protect subject confidentiality: Please see section IIF, "social risks" above.

E. Quality Assurance / Monitoring

All study staff will be trained to promote standardized and objective collection and recording of participant information. "Logic checks" are built into the CATSystem and into study data sheets to help identify and prevent inaccurate data entry. The US-based study manager will work with site coordinators on a quarterly basis to review data for accuracy, consistency, and completeness. Any missing or inconsistent data will be cross-checked with the hospital's paper-based records.

V. Data Analysis and Reporting

A. Statistical and Data Analysis: To demonstrate the efficacy of CATSystem verses SOC to increase the odds of women with a positive screen receiving treatment, we will model odds of treatment as a function of study arm and selected covariates (e.g., age, partner support, HIV status) using a logistic mixed model with a random site intercept included to adjust for clustering. Between-within degrees of freedom specified to adjust for downward bias in standard error estimates when the number of clusters is not large.82, 102 Matching of sites prior to randomization will improve balance between arms on site-level characteristics. Inclusion of covariates in the model will provide additional control for between-arm differences. We will use propensity score weighting to adjust for any severe, unanticipated imbalance between arms.

In post hoc analyses we will explore baseline patient and facility-level characteristics as potential correlates and moderators of the effect of CATSystem. Effects of these characteristics will be assessed by adding main effect and study arm×characteristic interaction terms to the logistic mixed model described above. We will also examine changes from baseline for key individual level factors [HIV status, partner support (see patient baseline survey in appendix)] for the two study arms. In further analyses, we will examine provider [level of experience, knowledge and motivation (see Provider survey in appendix)] as a mediator of the intervention's effect on odds of receiving treatment by fitting two models for each domain score: (1) A generalized or linear mixed model with change in

knowledge from baseline to end of study as outcome and study arm as explanatory variable, and (2) a log-binomial mixed model with receipt of treatment as (binary) outcome and change in provider knowledge from baseline to end of study as explanatory variable. Estimates from these models will be combined to assess direct and indirect effects of the intervention. We will similarly assess provider motivation, participant knowledge, and participant motivation as potential mediators.

Aim 2 analyses: FGDs will be digitally recorded and notes taken with participants' permission. Audio files will be translated and transcribed, coded, and analyzed using Dedoose, a qualitative data analysis software program. Transcriptions will be "open-coded": identifying key words and themes driven by the 14 specific TDF domains nested within the categories of Capability, Motivation, and Opportunity and provider recommendations for optimal implementation within existing health services. We will develop a codebook with typical exemplars for each theme, calculating the frequency and distribution of themes within larger topic areas. Two coders will establish inter-rater reliability and a third coder to settle discrepancies. Demographic and individual data will be summarized with descriptive statistics.

Aim 3 analyses: We will assess the cost-effectiveness of the CATSystem by evaluating how it impacts overall health system and societal costs and health outcomes as measured Quality-Adjusted Life-Years (QALYs). We will adhere to the Panel on Cost-Effectiveness guidelines in Health & Medicine109 and the ISPOR-SMDM Modeling Practices.110 We will track resource utilization during the trial (Yrs 2 and 3) and break out cost of the CATSystem intervention as well as follow-on screening and treatment costs in both the intervention and SOC arms. We will track screening, detection, and treatment rates from the trial. We anticipate the CATSystem will lead to higher screening costs, but may lead to lower cancer treatment costs (due to treatment of earlier stages being less expensive), lower health burden, and increased survival. Because early treatment may lead to fewer cancers in the long term, those "short-term" outcomes will then be translated into long-term outcomes using a mathematical model of cervical cancer disease. Using data from the trial and from the literature, we will track productivity as well including time (and transportation) to get screened, and productivity from cancer treatment.

B. **Outcome**: The primary outcome of SA 1 will be receipt of appropriate treatment (onsite or referral, as indicated) for clients with a positive screen. Figure 5 illustrates treatment scenarios for women with a positive screen and the targeted timeframe for treatment. Women with coinfections (cervicitis/STI) must receive treatment, resolution, and rescreening within 3 months to determine infection status and appropriate action. Patients with a positive screen for precancerous lesions should ideally receive same day onsite treatment with cryotherapy (max duration of 5 days/1 wk) or referral (in/outside facility) to LEEP for advanced lesions (w/in 5 days/1 wk) with treatment follow-up and negative rescreen. If suspected of invasive CC, a referral to KNH or MTRH for chemo/ radiotherapy within the month.

We will identify women presenting with precancerous lesions (mild, moderate or severe), suspicious of invasive CC, or those with unreliable screen results due to cervicitis/STI or other comorbidity in each arm. We will then determine if guideline adherent treatment was provided based on severity of screening results. Retention time from first screening to last follow-up will also be calculated and compared by arm. Patient and facility-level factors, including aggregated provider characteristics, will be assessed for their moderating impact on receipt of appropriate treatment after a positive screen. To

assess pathways through which the CATSystem works, we will measure provider and patient information, knowledge and motivation as potential mediators pre- and post-intervention across arms.

Aim 1 Secondary Outcomes: We will determine whether each participant is screened for CC during the intervention period and use these data to assess the effect of the CATSystem intervention on odds of screening. To adjust for sites' pre-intervention screening rates, we will compute the proportion of women screened for CC cancer during the pre-intervention period and include this variable as a covariate in modeling.

Aim 2 Outcomes: We will use the TDF framework and elements of human centered design to develop quantitative and qualitative questions that elicit feedback from intervention users (providers and patients) to gain a deep understanding of contextual factors that may act as barriers and/or facilitators to system use, and to assess CATSystem feasibility and acceptability. We will conduct brief surveys followed by focus group discussions (FGD) with providers interacting with the CATSystem to assess barriers and facilitators to: 1) provider implementation of CATSystem, 2) patient engagement with the system, and we will 3) identify strategies for optimal CATSystem implementation given provider capacity, existing systems, and patient preferences from a strengths based perspective. After implementation begins, we will also conduct surveys and FGDs with women enrolled in CATSystem who had a positive screen to assess users experience from the patient perspective.

C. **Study results to participants**: Findings regarding the impact of the intervention will be communicated with key stakeholder groups near study completion, including representatives from the patient and provider population.

D. **Publication Plan**: We plan to disseminate results of this study to all key stakeholders, including hospital, county, and national level health administrators and policy makers in Kenya; researchers and programmers, globally; and the community of caregivers of women in Kenya. We have set aside funds to present findings at national and international conferences, publish manuscripts in open access journals, and to support dissemination meetings within Kenya to ensure that local, county and national level stakeholders (including patients, clinicians, administrators and policy makers) are informed of key results of the research.

VI. Bibliography / References / Literature Cited

1. Black E, Richmond R. Prevention of Cervical Cancer in Sub-Saharan Africa: The Advantages and Challenges of HPV Vaccination. *Vaccines (Basel).* 2018;6(3):61.
2. Bateman LB, Blakemore S, Koneru A, et al. Barriers and Facilitators to Cervical Cancer Screening, Diagnosis, Follow-Up Care and Treatment: Perspectives of Human Immunodeficiency Virus-Positive Women and Health Care Practitioners in Tanzania. *The oncologist.* Jan 2019;24(1):69-75.
3. Ministry of health Kenya. National Cancer Control Strategy 2017-2022. Nairobi,June 2017.
4. Ng'ang'a A, Nyangasi M, Nkonge NG, et al. Predictors of cervical cancer screening among Kenyan women: results of a nested case-control study in a nationally representative survey. *BMC public health.* Nov 7 2018;18(Suppl 3):1221.
5. Finocchario-Kessler S, Gautney B, Cheng A, et al. Evaluation of the HIV Infant Tracking System (HITSystem) to optimise quality and efficiency of early infant diagnosis: a cluster-randomised trial in Kenya. *The Lancet HIV.* 2018/10/08/ 2018.
6. World Health Organization. Cervical Cancer: Key Facts. 2022; https://www.who.int/news-room/fact-sheets/detail/cervical-cancer#:~:text=Cervical%20cancer%20is%20the%20fourth,%2Dincome%20countries%20(1). 12/12/22.

7. Ferlay J, Ervik M, Lam F, et al. Global Cancer Observatory: Cancer Today. Lyon: International Agency for Research on Cancer; 20182018.
8. Bruni L B-RL, Albero G, Serrano B, Mena M, Gómez D, Muñoz J, Bosch FX, de Sanjosé S. *ICO/IARC Information Centre on HPV and Cancer (HPV Information Centre). Human Papillomavirus and Related Diseases in Kenya. Summary Report 27 July 2017.*2017.
9. ICO/IARC HPV Information Center. Kenya: Human Papillomavirus adn Related Cancers, Fact Sheet 2018. 2018; https://hpvcentre.net/statistics/reports/KEN_FS.pdf. Accessed April 30, 2021.
10. Denslow SA, Rositch AF, Firnhaber C, Ting J, Smith JS. Incidence and progression of cervical lesions in women with HIV: a systematic global review. *International journal of STD & AIDS.* 2014;25(3):163-177.
11. Kelly H, Weiss HA, Benavente Y, et al. Association of antiretroviral therapy with high-risk human papillomavirus, cervical intraepithelial neoplasia, and invasive cervical cancer in women living with HIV: a systematic review and meta-analysis. *The Lancet HIV.* 2018/01/01/ 2018;5(1):e45-e58.
12. Liu G, Sharma M, Tan N, Barnabas RV. HIV-positive women have higher risk of human papilloma virus infection, precancerous lesions, and cervical cancer. *Aids.* Mar 27 2018;32(6):795-808.
13. Coleman JS, Cespedes MS, Cu-Uvin S, et al. An insight into cervical cancer screening and treatment capacity in sub-Saharan Africa. *Journal of lower genital tract disease.* 2016;20(1):31.
14. Finocchario-Kessler S, Wexler C, Maloba M, Mabachi N, Ndikum-Moffor F, Bukusi E. Cervical cancer prevention and treatment research in Africa: a systematic review from a public health perspective. *BMC women's health.* 2016;16(1):29.
15. Kimani F, Sharif S, Bashir I. Ministry of Public Health and Sanitation and Ministry of Medical Services National Cervical Cancer Prevention Program in Kenya: Strategic Plan 2012-2015. Nairobi. *Nairobi, Kenya.* 2012.
16. Khozaim K, Orang'o E, Christoffersen-Deb A, et al. Successes and challenges of establishing a cervical cancer screening and treatment program in western Kenya. *International journal of gynaecology and obstetrics: the official organ of the International Federation of Gynaecology and Obstetrics.* Jan 2014;124(1):12-18.
17. Orang'o EO, Wachira J, Asirwa FC, et al. Factors associated with uptake of visual inspection with acetic acid (VIA) for cervical cancer screening in Western Kenya. *PloS one.* 2016;11(6):e0157217.
18. Rohner E, Bütikofer L, Schmidlin K, et al. Cervical cancer risk in women living with HIV across four continents: A multicohort study. *International Journal of Cancer.* 2020;146(3):601-609.
19. Brotherton J, Davies C, Committee IP. IPVS policy statement. Equity in cervical cancer prevention: for all and not just for some. *Papillomavirus Res.* 2020;9:100192-100192.
20. Williams-Brennan L, Gastaldo D, Cole DC, Paszat L. Social determinants of health associated with cervical cancer screening among women living in developing countries: a scoping review. *Archives of gynecology and obstetrics.* 2012;286(6):1487-1505.
21. Hurt K, Walker RJ, Campbell JA, Egede LE. mHealth interventions in low and middle-income countries: a systematic review. *Global journal of health science.* 2016;8(9):183.
22. Mariwa F. Internet Access: How Rural Kenya is keeping pace. 2019; https://www.dw.com/en/internet-access-how-rural-kenya-is-keeping-pace/a-47071209. Accessed April 4, 2021.
23. Nitsche. L. Mobile Solutions a catalyst for Internet penetration in Kenya. 2019; https://www.dw.com/en/mobile-solutions-a-catalyst-for-internet-penetration-in-kenya/a-47078206. Accessed May 3, 2021.
24. Gibson DG, Ochieng B, Kagucia EW, et al. Mobile phone-delivered reminders and incentives to improve childhood immunisation coverage and timeliness in Kenya (M-SIMU): a cluster randomised controlled trial. *The Lancet Global Health.* 2017;5(4):e428-e438.
25. Wahl B, Cossy-Gantner A, Germann S, Schwalbe NR. Artificial intelligence (AI) and global health: how can AI contribute to health in resource-poor settings? *BMJ global health.* 2018;3(4):e000798.
26. Jawhari B, Ludwick D, Keenan L, Zakus D, Hayward R. Benefits and challenges of EMR implementations in low resource settings: a state-of-the-art review. *BMC Medical Informatics and Decision Making.* 2016/09/06 2016;16(1):116.

27. Nandi County Ministry of Health. *Nandi County HIV & AIDS Strategic Plan 2015/2016-2018/2019.* Nandi County Kenya: Nandi ounty Ministry of Health;2018.
28. Bungoma County Ministry of Health. *Bungoma County HIV & AIDS Strategic Plan 2014/2015-2018/2019.* Bungoma Kenya: Bungoma County Ministry of Health;2018.
29. Muttai H, Guyah B, Achia T, et al. Mapping geographic clusters of new HIV diagnoses to inform granular-level interventions for HIV epidemic control in western Kenya. *BMC Public Health.* 2021/10/23 2021;21(1):1926.
30. Proctor E, Silmere H, Raghavan R, et al. Outcomes for implementation research: conceptual distinctions, measurement challenges, and research agenda. *Adm Policy Ment Health.* Mar 2011;38(2):65-76.
31. Weiner BJ, Lewis CC, Stanick C, et al. Psychometric assessment of three newly developed implementation outcome measures. *Implementation Science.* 2017/08/29 2017;12(1):108.
32. Leyrat C, Morgan KE, Leurent B, Kahan BC. Cluster randomized trials with a small number of clusters: which analyses should be used? *Int J Epidemiol.* Feb 1 2018;47(1):321-331.
33. Staggs VS, Feldman K. Use of between-within degrees of freedom as an alternative to the Kenward–Roger method for small-sample inference in generalized linear mixed modeling of clustered count data. *Communications in Statistics - Simulation and Computation.* 2021:1-11.
34. Vanderweele TJ, Vansteelandt S. Odds ratios for mediation analysis for a dichotomous outcome. *Am J Epidemiol.* Dec 15 2010;172(12):1339-1348.
35. Turner RM, Omar RZ, Thompson SG. Bayesian methods of analysis for cluster randomized trials with binary outcome data. *Statistics in Medicine.* 2001;20(3):453-472.
36. Seaman SR, White IR. Review of inverse probability weighting for dealing with missing data. *Stat Methods Med Res.* Jun 2013;22(3):278-295.
37. Amrhein V, Greenland S, McShane B. Scientists rise up against statistical significance. *Nature.* Mar 2019;567(7748):305-307.
38. Wasserstein RL, Schirm AL, Lazar NA. Moving to a World Beyond “p < 0.05”. *The American Statistician.* 2019/03/29 2019;73(sup1):1-19.
39. Betensky RA. The p-Value Requires Context, Not a Threshold. *The American Statistician.* 2019/03/29 2019;73(sup1):115-117.

APPENDIX I: VULNERABLE POPULATIONS

I. **Cognitively or decisionally impaired individuals**: Cognitively or decisionally impaired individuals will not be eligible for participation

II. **Children**: Girls between the ages of 16 and 18 are eligible for inclusion in this study, with consent from a legal guardian and assent from the girl. RAs will take additional time with these young participants to ensure comprehension, emphasizing that participation is optional and is not a requirement for receiving standard of care cervical cancer screening and treatment services. For girls participating in the study who attain 18 years while in the study, re-consent will be conducted at the first clinical presentation after their 18th birthday. If consent is not attained at during this re-consent process, the woman will be withdrawn and her data from <18 years will be excluded from study analyses.

III. **Pregnant women:** Per Kenyan guidelines, women above 20 weeks gestation are ineligible for cervical cancer screening; thus, pregnant women above 20 weeks gestation will be excluded from enrollment in the study. If a previously enrolled woman reaches 20 weeks gestation, she will continue to be tracked by study procedures, including CATSystem follow up so that treatment can be initiated post-delivery.

IV. Prisoners: N/A

V. Students and/or Employees: N/A
